# Exploring Factors Affecting the Emergency Specialists' Decision-Making in Case of Emergencies in Patients

**DOI:** 10.1155/2018/9579807

**Published:** 2018-05-17

**Authors:** Abbasali Ebrahimian, Seyed-Hossein Hashemi-Amrei, Mohammadreza Monesan

**Affiliations:** ^1^Nursing Care Research Center, Semnan University of Medical Sciences, Semnan, Iran; ^2^Emergency Nursing Group, Nursing and Midwifery School, Semnan University of Medical Sciences, Semnan, Iran; ^3^Student Research Committee, Semnan University of Medical Sciences, Semnan, Iran; ^4^Department of Emergency Medicine, Kowsar Educational and Research Center, Semnan University of Medical Sciences, Semnan, Iran

## Abstract

**Introduction:**

Appropriate decision-making is essential in emergency situations; however, little information is available on how emergency decision-makers decide on the emergency status of the patients shifted to the emergency department of the hospital. This study aimed at explaining the factors that influence the emergency specialists' decision-making in case of emergency conditions in patients.

**Methods:**

This study was carried out with a qualitative content analysis approach. The participants were selected based on purposive sampling by the emergency specialists. The data were collected through semistructured interviews and were analyzed using the method proposed by Graneheim and Lundman.

**Results:**

The core theme of the study was “efforts to perceive the acute health threats of the patient.” This theme was derived from the main classes, including “the identification of the acute threats based on the patient's condition” and “the identification of the acute threats based on peripheral conditions.” *Conclusions*. The conditions governing the decision-making process about patients in the emergency department differ from the conditions in other health-care departments at hospitals. Emergency specialists may have several approaches to decide about the patients' emergency conditions. Therefore, notably, the emergency specialists' working conditions and the others' expectations from these specialists should be considered.

## 1. Introduction

Appropriate decision-making is essential in emergency situations [1]. The emergency medical services (EMS) system is one of the systems where crucial emergency decisions are taken [2]. In this system, the EMS staff are often recognized as the first medical service providers [3]. Due to their presence during the incident, they are completely aware of the emergency condition [4]. They make key decisions in prehospital settings regarding the onset of treatment, prioritization of the tasks required for shifting patients, and the appropriate department where the patient needs to be shifted [3]; however, controversies exist in certain cases considering the emergency of the patients' circumstances among the EMS staff and emergency specialists in hospitals. Such controversies among different medical groups with emergency specialists are common and have been addressed in certain studies. For example, in a study, two groups of emergency physicians and family physicians were requested to categorize 17 patients hospitalized in the emergency department whether or not they need to be transmitted by ambulance [5]. The results of this study reported an overlap in only 20% of the opinions between the two groups of the physicians [6]. The results of another study reported that 28.9% of patients with internal diseases shifted by the prehospital emergency staff did not require emergency transfer according to the emergency specialists, and 21% of the patients were not so severely affected to be shifted to the hospitals [4]. Other research findings reported that the opinions of the EMS staff and emergency specialists regarding the need for the transferring 1330 patients with internal diseases by ambulance were similar only in 33.23% of the cases [7]. Moreover, Challen and Walter reported that only 65% of the 215 patients who were recognized as eligible by EMS for hospital transfer were admitted to the hospital emergency department [5].

The explanation of the decision-making styles about the emergency patients by various medical and paramedical groups is one of the methods by which it is possible to reach a consensus on the emergency or nonemergency state of the patients referring to the hospital emergencies. One of the studies in this context has attempted to explain the effective background factors in the prehospital emergency staff's decision-making regarding the transfer of patients with internal diseases from the prehospital environment [8]. The results of this study reported that the EMS staff's decisions are not made solely based on the patients' physical condition, but other factors as well, such as the patient's socioeconomic and cultural status, the conditions of the mission, and the characteristics of the EMS staff [9]; however, little is known on how the emergency decision-makers decide on the emergency status of the patients shifted to the emergency department of the hospital. Presumably, explaining how the emergency decision-makers decide on the emergency status of the patients can help with the expansion of the concept of the emergency circumstances of patients from the perspective of emergency specialists. A clear view of this concept will lead to a better understanding of the opinions of the emergency specialists by other EMS specialists and staff and, subsequently, to a similar approach between them. Therefore, this study was conducted with an objective to explain the factors that influence the emergency specialists' decision-making regarding the emergency condition of patients.

## 2. Methods

### 2.1. Study Design and Settings

This study was carried out with a qualitative content analysis approach in 2016. The data were collected individually by the researcher through semistructured interviews. Before conducting the interviews, in order to observe the principles of ethics in research, the researcher introduced himself to the participants, showed the ethics committee's permission to the participants, and explained the research objectives to them. Thereafter, the specialists who were willing to cooperate with the researcher completed the informed consent form. After the completion of this form, the time and location of the interviews were decided with the direct opinions of the participants. In fact, in coordination with the participants, all the interviews were conducted in Imam Khomeini Hospital in Sari and Kowsar Hospital in Semnan.

### 2.2. Participants

The participants were selected based on purposive sampling from the emergency specialists working in the hospitals affiliated to Mazandaran and Semnan Universities of Medical Sciences. The criteria for inclusion in this study involved complete knowledge of the phenomenon under the study, at least 1-year experience of working in the hospital emergency department, and willingness for the transfer of experiences.

### 2.3. Data Gathering

**Figure 1:**
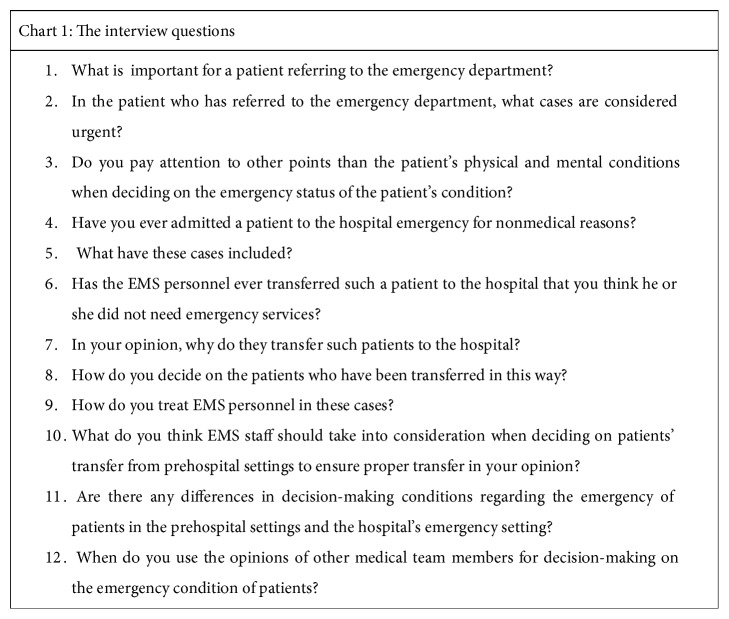
The interview questions.

In total, 14 emergency specialists were interviewed until data saturation was reached. Notably, for the interview, 9 participants selected the time before their working shift and 5 of them selected the time after their working shift. The interviews commenced with a general question: what is important for a patient referring to the emergency department? Thereafter, the interviews proceeded with the supplementary questions quoted in Figure 1. In addition, exploratory questions were also used to better understand the interviewees' experiences. At the end of the interviews, the participants were asked to speak freely in case of other points regarding the interview. All the interviews were recorded using a digital recorder after taking the participants' permission and were transcribed into a written text immediately. The interviews continued until the data reached saturation.

### 2.4. Statistical Analysis

Data were analyzed after conducting the first interview. The data quality management software program, MAXQDA 2010, was used to manage the data. The data obtained from the interviews were analyzed using the method proposed by Graneheim and Lundman. According to this method, the researcher first reads the text of the interviews several times from the beginning to the end in order to acquire enough knowledge about the data. In this study, the entire text of each interview was considered as a unit of analysis and the words, sentences, or paragraphs were considered as meaning units. Moreover, the meaning units were coded using the expressions of interviewees or new concepts. In the next step, the codes were compared in terms of similarities and differences and were classified into more abstract classes. Eventually, the content latent in the data was introduced as the main theme of the study by comparing the classes and deep reflection on them [10]. After analyzing each interview in terms of the credibility of the data, the interview text along with the initial extracted codes was given to the interviewee. At this stage, the interviewee was asked to read the researcher's analyses and declare if there is any difference between the researcher's analysis and the participant's point of view. In cases where the participant's opinion was different from the researcher's analysis, the concept was corrected with the help of the participant. In addition, the analysis process, extraction of codes, and the method to access the subclasses, classes, and the main theme of the study were discussed, reviewed, and confirmed in presence of two other researchers [11].

## 3. Results

It is noteworthy that 12 men and 2 women with the mean age of 43.07 ± 5.70 years and work experience of 8.00 ± 3.41 years participated in this study. Among the participants, 11 experts were employed in the Emergency Department of Imam Khomeini Hospital in Sari and 3 experts were employed in the Emergency Department of Kowsar Hospital in Semnan.

The main theme of the study was “efforts to perceive the acute health threats of the patient.” This objective was representative of the main approach of emergency specialists when making decision about the patients' conditions in the emergency department of the hospital. This objective was derived from the main classes, including “the identification of the acute threats based on the patient's condition” and “the identification of the acute threats based on peripheral conditions” (Table 1). These classes and the related subclasses are described below.

### 3.1. Identification of the Acute Threats Based on the Patient's Condition

All the emergency medical experts participating in the present study attempted to identify the acute health threats of patients based on their recent status. They tried to analyze the patients' conditions based on the scientific resources, clinical experiences, and diagnostic procedures.

### 3.2. Clinical Conditions

Many participants tried to adapt the signs and symptoms of patients to the content of the scientific literature and to estimate the extent of patients' health threats accordingly. In addition, most of the participants used the designated triage level for patients as a scientific index contributing to the identification of patients' health threats.

#### 3.2.1. Participant 1

The patient, who is in shock, has a low level of consciousness, has low GCS, and has unstable vital signs, is an emergency patient.

#### 3.2.2. Participant 8

Based on the triage level, it becomes clear what the patient's general state is. The critically ill patients are categorized in levels 1 and 2, and the other patients are categorized in levels 3 and 4.

#### 3.2.3. Participant 13

As a whole, patients' vital conditions are of utmost importance to me; accordingly, these individuals are referred to triage mission and their vital signs are checked considering their conditions.

### 3.3. Clinical Experiences

The participants assumed that comparing the patient's condition with the conditions of the patients with whom they have been previously in contact is helpful in detecting the risks that threaten patients' health. Similarly, some participants said that their previous experiences make us doubt the state of the patient with no apparent serious problem. In such situations, the decision about the acute condition of the patient is made based on suspicion, and the subsequent actions are taken with higher caution and carefulness. Such patients are deemed to be in an emergency situation until no health threat is completely ensured. Most of the participants used the patient's response to treatment measures in the emergency department as a criterion to diagnose the acute conditions of the patient. For example, the health of the anesthetized patient who responds to the first naloxone or the first glucose vial is at a lower risk compared to the one that does not respond appropriately to the emergency measures.

#### 3.3.1. Participant 4

Experience has revealed that all the conditions of the patients who are transferred to the emergency department after trauma and crash should be recorded in the patient's file. Furthermore, it should be checked and announced whether they have certain problems. Then, the patient should be discharged.

#### 3.3.2. Participant 11

The patient with a high level of sugar in his or her blood should stay in the emergency room for the regulation of an insulin dose. Therefore, the emergency status depends upon the response to the treatment.

#### 3.3.3. Participant 5

Patients with a history of cancer may now refer with swollen necks, and when their neck is checked, subcutaneous emphysema can be felt. Experience has also shown that these individuals are likely to be suffering from esophageal lesions and chips, so they can be considered as major medical emergencies.

### 3.4. Additional Diagnostic Methods

Almost all the participants used the results of the diagnostic tests and consultation with other professionals as a certain method to diagnose the deterioration of patients' diseases. They usually took consultation and advice from other professionals in situations where the patients' status was complicated or the patients required more specialized care to continue their treatment.

#### 3.4.1. Participant 14

Sometimes, I cannot relieve the patient's mind with a very detailed examination. I tell him or her that I should also give a test, I also need to do an ultrasound, I need to take some paraclinical measures, and then, I will tell him or her that he or she can go without any preoccupation.

#### 3.4.2. Participant 3

For example, when a patient refers, with a change in the state of consciousness, we check his or her sugar level, inject a naloxone, and conduct a series of tests, a CT scan, and LP. By these measures, we may or may not diagnose the issue.

#### 3.4.3. Participant 1

Sometimes, it is so complicated to analyze the patients' conditions that there is a need to consult with the relevant specialists to diagnose the disease.

### 3.5. Identification of Acute Threats Based on Peripheral Conditions

A large number of emergency specialists took into consideration the peripheral conditions of the patients when deciding on the urgency of the patients' condition in addition to paying attention to the patients' signs and symptoms. These conditions included the patients' conditions before arrival to the emergency department, work considerations, and patients and their families' approaches.

### 3.6. Patients' Conditions before Arrival to the Emergency Department

Despite the fact that the patients' conditions in the hospital emergency room were reevaluated based on their clinical status, the emergency specialists were keen to know what the patients' conditions were before they were transferred to the hospital emergency room, and to know what measures have been taken toward the patients by the EMS technicians or other people present on the scene. From the viewpoint of the emergency specialists, the measures taken prior to the transfer of the patients to the emergency department were divided into three categories, that is, useful, harmful, and neutral measures. The useful measures were the actions that reduced the severity of injuries to patients or prevented the progress of injuries. The harmful measures were the ones that led to disease progression or imposed more harm to the patient. The neutral measures were also the ones that did not do any good to the patient. They asked these questions to the patients themselves, EMS staff, patients' relatives, or other people who had transferred the patients to the hospital. These conditions often influenced the specialists' decisions. If the specialists realized that the measures taken prior to the patients' transmission to hospital were harmful, they would estimate the severity of the emergency of patients' conditions to be higher.

#### 3.6.1. Participant 7

Occasionally, the EMS staff does not follow an appropriate approach. For example, naloxone should be injected into the patient with narcotic toxicity when the patient's breathing rate is disrupted; however, if naloxone is injected into the patient when not required, we should monitor him or her for 6 h.

#### 3.6.2. Participant 2

If the patient has a dislocation that disrupts the circulation (such as knee dislocation that may harm the popliteal artery), it will often be fixed on the scene and will prevent further damage.

#### 3.6.3. Participant 9

Knowing about patients' previous conditions, especially in ones who have suffered from unconsciousness and been unable to speak, is effective in determining the severity of the patients' conditions. For example, we need to know what factors have been the causes of patients' consciousness.

### 3.7. Work Conditions

Almost all emergency staff members considered several factors when deciding on the status of patients. Of course, these considerations differed in terms of the specialists' personality and scientific and clinical capabilities. The most serious consideration pertained to the patient's legal issues, professional rules and regulations, and specific hospital rules and regulations. Similarly, the availability of sufficient facilities for the specialists would allow them to pay excessive attention to some patients. For example, when there was no portable CT scan device in the department, the specialists considered a patient suspected of intracranial hemorrhage as a high-risk patient until the CT scan would be carried out at the CT scan center of the hospital.

#### 3.7.1. Participant 5

Occasionally, the patient will need his or her file later; for example, he or she needs to have a file in the emergency department in sexual crimes. In this case, his or her condition is recorded in the file so that it can be commented in the future whether he or she has had any problem or not based on the file.

#### 3.7.2. Participant 13

When we need more technical diagnostic tools, our decisions are not comparable to when we have limited equipment.

#### 3.7.3. Participant 10

When there is a doubt whether or not a patient has had a brain hemorrhage, the patient receives intensive care until the CT scan results are reported.

### 3.8. Patients and Families' Approaches

Patients and their families could also be involved in the decision-making of the emergency specialists. This role comprised two concepts regarding the expectations of patients and their families and the perceived level of the disease severity by the patient and his or her family. Such factors including the emergency level of the patient and family awareness of the present facilities of the hospital and the level of patients' expectation of the health system were effective in shaping the concept of the patients and their families' expectations. In addition, the level of patients and their family awareness of the problem, the patients and their family's hope of complete or partial improvement, the level of general literacy, and the level of medical knowledge were effective in the degree of the perceived level of the disease severity by the patient and his or her family.

#### 3.8.1. Participant 4

In determining the severity of the patients' emergency, other than the signs and symptoms that we observe in the patient, the patient himself or herself is also involved, that is, the severity of an emergency depends on how much the patient feels to be in an emergency state. That is, the patient's words are also important, that is, he or she regards himself or herself as an emergency patient.

#### 3.8.2. Participant 6

More often, there is no need for the patient transfer; indeed, the patient needs only clinical measures, but the patient's expectation is to get hospitalized. In such cases, they call the EMS and the EMS will have to transfer the patient to the hospital.

#### 3.8.3. Participant 14

The cultural range of those referring to the emergency department is very different. There are a series of people who know what an emergency department has been made for, and if there is no emergency, the patients are not taken to this department. Some people also take patients with nonemergency conditions to the hospital and urge us to provide emergency care. In some cases, this can challenge our decisions.

## 4. Discussion

This study aimed to explain the factors influencing the emergency specialists' decision-making regarding the emergency status of patients' conditions. The findings of this study led to the emergence of a major research objective and two main classes. The objective of the study was representative of the main focus on how emergency decision-makers decided on the emergency conditions of the patients. In this study, factors such as clinical conditions, clinical experiences, diagnostic procedures, prearrival conditions to the emergency department, work considerations, and patients and their families' approaches were shaped around the axial factor, and it was reported what parameters are taken into consideration by an emergency specialist at the time of the decision-making about the emergency conditions of patients.

The consideration of the patients' clinical conditions played a crucial role in the emergency specialists' decision-making regarding the emergency of the conditions of the patient referring to the emergency department. The presence of clinical signs and symptoms and the level of triage determined based on ESI were the most important criteria for deciding upon the emergency conditions of patients by emergency specialists. In some studies, the presence of unnatural clinical signs and symptoms has been crucial in assessing the severity of the patient's disease. Ebrahimian et al. carried out a study to identify the factors affecting the EMS staff's decision-making of the transfer of patients with internal diseases. In that study, they reported that attention to the physical condition of patients with internal diseases played an important role in the EMS staff's decisions about the transmission of these patients to the internal wards [9]. Frost and Weise recognized symptoms such as seizure, coma, tachycardia, bradycardia, dizziness, hypotension, cyanosis, coldness of the external body environment, tachypnea, bradypnea, restlessness, and urinary retention as the patients' risky clinical signs and symptoms [8]. This indicates that the emergency specialists' decision-making is more based on the patients' signs and symptoms and the type of the disease is not that much involved in decision-making. In fact, emergency specialists' decisions are more “symptomatic” and “symptom-based.”

Another subclass of this study was “clinical experiences.” This subclass included three concepts of “similar clinical conditions,” “the recognition of the suspected emergency situations based on experience,” and “patient response rates to the already-taken emergency measures.” Emergency specialists have read the principles of patient treatment in their various reference books; however, clinical experiences make it possible for them to apply their theoretical knowledge in a practical environment. By practicing theoretical knowledge, they achieve a unique level of medical science as a clinical experience that helps them use new and more effective pathways to treat patients according to their conditions. The rate of their success in creating new scientific pathways and achieving clinical experience is directly related to their new understanding of the learned materials. In a study carried out at Christchurch Hospital in New Zealand, Than et al. reported that the use of an experimental pathway in the emergency department of hospitals is more effective in identifying the actual patients with acute coronary syndrome compared with the use of a standardized pathway [12]. In addition, emergency specialists can also help with the academic development of their discipline by sharing their personal experiences. Delir-Haghighi et al. reported that the lack of staff's personal experiences in the emergency department is one of the major problems in decision-making in this department. They believed that these experiences should be collected, stored, and shared to make clever decisions in emergency departments [13].

Emergency specialists, like specialists in other disciplines, also used “diagnostic procedures” to make decisions about their patients. They usually sought help from the paraclinical tests and through consultation with specialists in other medical disciplines when they could not take appropriate decisions about patients using their own knowledge and experience. Most of the participants believed that the use of paraclinical tests would greatly help with the better understanding of the patients' status. For this reason, they were interested in requesting for a large number of paraclinical tests for patients in a short time. In this regard, Griffey et al. reported that the emergency physicians overused CT scan to determine the conditions of patients [14]. Presumably, the reason for the overuse of diagnostic procedures in emergency departments is the physicians' high sensitivity to understand the underlying cause of the disease in a short time. Several clients referring to the emergency department were busy with their normal life a few hours before being transferred to the emergency department. Therefore, patients, their families, and the medical team attempt with high sensitivity to identify and resolve the causes of acute health threats in a short period of time.

“Patients' conditions before arrival to the emergency department” was also effective in the emergency specialists' decision-making regarding the emergency conditions of the patients. This factor comprised two components, namely, the quality of the services provided before the patient's arrival to the emergency department and the triage level reported by the EMS staff. In several studies, it has also been suggested that the early warning system is used to determine the patients' status before arrival to the emergency department of the hospital [15, 16]. The comparison of the conditions prior to the patients' arrival to the emergency department with their present conditions can help the specialists make better decisions. For example, the patient who was at level 3 of triage prior to transfer and has been at level 2 of triage after being admitted to the emergency department has a worse clinical condition than the patient who has reached from level 3 of triage to level 4.

The breakdown or unavailability of the necessary equipment also affected the emergency specialists' decision-making regarding the patients' emergency conditions. Ebrahimian et al. also reported that the lack of diagnostic facilities affects the judgment of the EMS staff [9]. Attention to the rules and guidelines was also an effective factor in the emergency specialists' decision-making. In fact, the consideration of rules and guidelines was the main criterion for making the final decisions about the patients' conditions.

One of the other effective components in specialists' decision-making was the “patients and their families' approaches.” The patients' cultural status, level of medical knowledge, degree of social maturity, and economic status made them have different expectations of the provision of health services in the emergency department. This could partly influence the specialists' decision-making. Ebrahimian et al. reported that the socioeconomic status and cultural status of patients are effective in decision-making on the emergency status of patients' conditions [9]. Some other studies have also reported that socioeconomic status is one of the determinants of health [17, 18]. Therefore, the emergency specialists' decision-making was partially influenced by the patients and their families' beliefs.

## 5. Conclusion

The explanation of the effective factors in the emergency specialists' decision-making on the emergency status of the patient's conditions helps the emergency specialists, other medical professionals, and other members of the medical team to better perceive the decision-making conditions in emergency departments. This study revealed that the conditions governing the decision-making process about patients in the emergency department differ from the conditions in other health-care departments at hospitals. While making a decision on the emergency of patients' conditions, the emergency specialists also pay attention to the patients' peripheral conditions in addition to the patients' physical issues and problems. They may consider different approaches to make decisions about the patients' emergency conditions based on their level of experience and responsibility; however, their main focus is to understand the acute health threats of patients. They should make critical decisions for patients in an unstable and stressful situation. Therefore, their decisions may be regarded as pessimistic from the perspective of other members of the medical team. Therefore, it is suggested that attention be paid to the emergency specialists' working conditions and the expectations others have regarding them when judging the correctness of their decisions.

## 6. Limitations

The specialists participating in this study had sufficient work experience in the emergency department of the hospital. They used their recent experiences when responding to the questions. Therefore, this study has encountered some limitations in terms of depicting the decision-making method of the inexperienced emergency specialists.

## Figures and Tables

**Table 1:** Factors affecting the emergency specialists' decision-making in case of emergencies in patients.

Main theme: efforts to perceive the acute health threats of the patient		
Categories	Subcategories	Concepts
Identification of the acute threats based on the patient's condition	Clinical conditions	Critical signs and symptoms
		Designated triage level for patients
	Clinical experiences	Similar clinical conditions
		Recognition of suspected emergency situations based on experience
		The patient's response to treatment measures
	Additional diagnostic methods	The results of diagnostic tests
		Consultation

Identification of acute threats based on peripheral conditions	Patients' conditions before arrival to the emergency department	The patient quality services before admitted the emergency department
		Triage level reported by EMS staff
	Work conditions	Sufficient or insufficient facilities
		Professional rules and regulations
	Patients and families' approaches	Expectations of patients and their families
		The perceived level of the disease severity by the patient and his/her family

## References

[1] Sun B., Ma W., Zhao H. (2015). An approach to emergency decision making based on decision-theoretic rough set over two universes. *Soft Computing*.

[2] Ebrahimian A., Khalesi N., Mohamadi G., Tordeh M., Naghipour M. (2012). Transportation management in pre-hospital emergency with physiological early warning scores. *Journal of Health Administration*.

[3] Fullerton J. N., Price C. L., Silvey N. E., Brace S. J., Perkins G. D. (2012). Is the Modified Early Warning Score (MEWS) superior to clinician judgement in detecting critical illness in the pre-hospital environment?. *Resuscitation*.

[4] Mulholland S. A., Gabbe B. J., Cameron P. (2005). Is paramedic judgement useful in prehospital trauma triage?. *Injury*.

[5] Challen K., Walter D. (2009). Physiological scoring: an aid to emergency medical services transport decisions?. *Prehospital and Disaster Medicine*.

[6] Mann C., Guly H. (1998). Is the emergency (999) service being misused? Retrospective analysis. *BMJ*.

[7] Ebrahimian A. A., Shabanikiya H. R., Khalesi N. (2012). The role of physiological scores for decision making in internal pre-hospital emergency situations. *HealthMED*.

[8] Frost P. J., Wise M. P. (2012). Early management of acutely ill ward patients. *BMJ*.

[9] Ebrahimian A., Seyedin H., Jamshidi-Orak R., Masoumi G. H. (2014). Exploring factors affecting emergency medical services staffs’ decision about transporting medical patients to medical facilities. *Emergency Medicine International*.

[10] Graneheim U. H., Lundman B. (2004). Qualitative content analysis in nursing research: concepts, procedures and measures to achieve trustworthiness. *Nurse Education Today*.

[11] Mayring P. H. (2000). Qualitative content analysis. *Forum: Qualitative Social Research*.

[12] Than M., Aldous S., Jane-Lord S. (2014). A 2-hour diagnostic protocol for possible cardiac chest pain in the emergency department: a randomized clinical trial. *JAMA Internal Medicine*.

[13] Delir-Haghighi P., Burstein F., Zaslavsky A., Arbonc P. (2013). Development and evaluation of ontology for intelligent decision support in Medical Emergency Management for mass gatherings. *Decision Support Systems*.

[14] Griffey R. T., Jeffe D. B., Bailey T. (2014). Emergency physicians’ attitudes and preferences regarding computed tomography, radiation exposure, and imaging decision support. *Academic Emergency Medicine*.

[15] Duckitt R. W., Buxton-Thomas R., Walker J. (2007). Worthing physiological scoring system: derivation and validation of a physiological early-warning system for medical admissions. An observational, population-based single-centre study. *British Journal of Anaesthesia*.

[16] Ebrahimian A., Masoumi G., Jamshidi-Orak R., Seyedin H. (2017). Development and psychometric evaluation of the pre-hospital medical emergencies early warning scale. *Indian Journal of Critical Care Medicine*.

[17] Pereira G. N., Bastos G. A. N., Del Duca G. F., Bós A. J. G. (2012). Socioeconomic and demographic indicators associated with functional disability in the elderly. *Cadernos de Saúde Pública*.

[18] Siracuse J. J., Odell D. D., Gondek S. P. (2012). Health care and socioeconomic impact of falls in the elderly. *American Journal of Surgery*.

